# Bridging Insights From Lymph Node and Synovium Studies in Early Rheumatoid Arthritis

**DOI:** 10.3389/fmed.2021.820232

**Published:** 2022-01-14

**Authors:** Aoife M. O'Byrne, Tineke A. de Jong, Lisa G. M. van Baarsen

**Affiliations:** ^1^Department of Rheumatology and Clinical Immunology, Amsterdam Institute for Infection and Immunity, Amsterdam University Medical Center, University of Amsterdam, Amsterdam, Netherlands; ^2^Department of Experimental Immunology, Amsterdam Institute for Infection and Immunity, Amsterdam University Medical Center, University of Amsterdam, Amsterdam, Netherlands; ^3^Amsterdam Rheumatology and Immunology Center (ARC), Amsterdam, Netherlands

**Keywords:** rheumatoid arthritis, lymph node, synovium, tolerance, fibroblasts, T cells, pre-RA

## Abstract

Rheumatoid arthritis (RA) is a chronic autoimmune disease of unknown etiology characterized by inflammation of the peripheral synovial joints leading to pannus formation and bone destruction. Rheumatoid Factor (RF) and anti-citrullinated protein antibodies (ACPA) are present years before clinical manifestations and are indicative of a break in tolerance that precedes chronic inflammation. The majority of studies investigating disease pathogenesis focus on the synovial joint as target site of inflammation while few studies explore the initial break in peripheral tolerance which occurs within secondary lymphoid organs such as lymph nodes. If explored during the earliest phases of RA, lymph node research may provide innovative drug targets for disease modulation or prevention. RA research largely centers on the role and origin of lymphocytes, such as pro-inflammatory T cells and macrophages that infiltrate the joint, as well as growing efforts to determine the role of stromal cells within the synovium. It is therefore important to explore these cell types also within the lymph node as a number of mouse studies suggest a prominent immunomodulatory role for lymph node stromal cells. Synovium and proximal peripheral lymph nodes should be investigated in conjunction with one another to gain understanding of the immunological processes driving RA progression from systemic autoimmunity toward synovial inflammation. This perspective seeks to provide an overview of current literature concerning the immunological changes present within lymph nodes and synovium during early RA. It will also propose areas that warrant further exploration with the aim to uncover novel targets to prevent disease progression.

## Introduction

Rheumatoid arthritis (RA) is a chronic inflammatory autoimmune disease of unknown etiology that preferentially affects the peripheral joints. It is characterized by prolonged inflammation of the synovium which eventually leads to tissue destruction and pannus formation. Current research indicates that this chronic inflammation is driven by the infiltration of destructive pro-inflammatory lymphocytes into the joint leading to pro-inflammatory cytokine release and the initiation of an immune response. This response is perpetuated by the interaction of these destructive immune cells e.g. macrophages and stromal cells present within the synovium. Autoantibodies like rheumatoid factor (RF) and anti-citrullinated peptide antibodies (ACPA) are present in the peripheral blood of RA patients up to a decade before the clinical onset of synovitis and diagnosis (RA-risk individuals) (1–4). This highlights a break in immune tolerance which results in systemic autoimmunity years before diagnosis. Furthermore, prospective studies showed that depending on the risk profile only 30% of individuals with autoantibodies go on to develop arthritis (5, 6), suggesting that additional genetic and environmental factors influence disease onset.

The current paradigm highlights three key phases of arthritis development: the initial break in tolerance, the infiltration of immune cells into the synovial joints and finally established chronic synovitis leading to joint destruction (7). Although much is known about the chronic inflammatory process occurring in the synovium at the latter stages of disease, the changes that occur in the immune system during the earliest phases when there is break in tolerance but no apparent synovitis have yet to be fully elucidated. This break in tolerance is hypothesized to occur in secondary lymphoid organs such as lymph nodes (LNs). Currently, only a few human studies have explored this, while it may provide major insights into early RA pathogenesis and reveal mechanisms for restoring peripheral tolerance.

**Figure 1:**
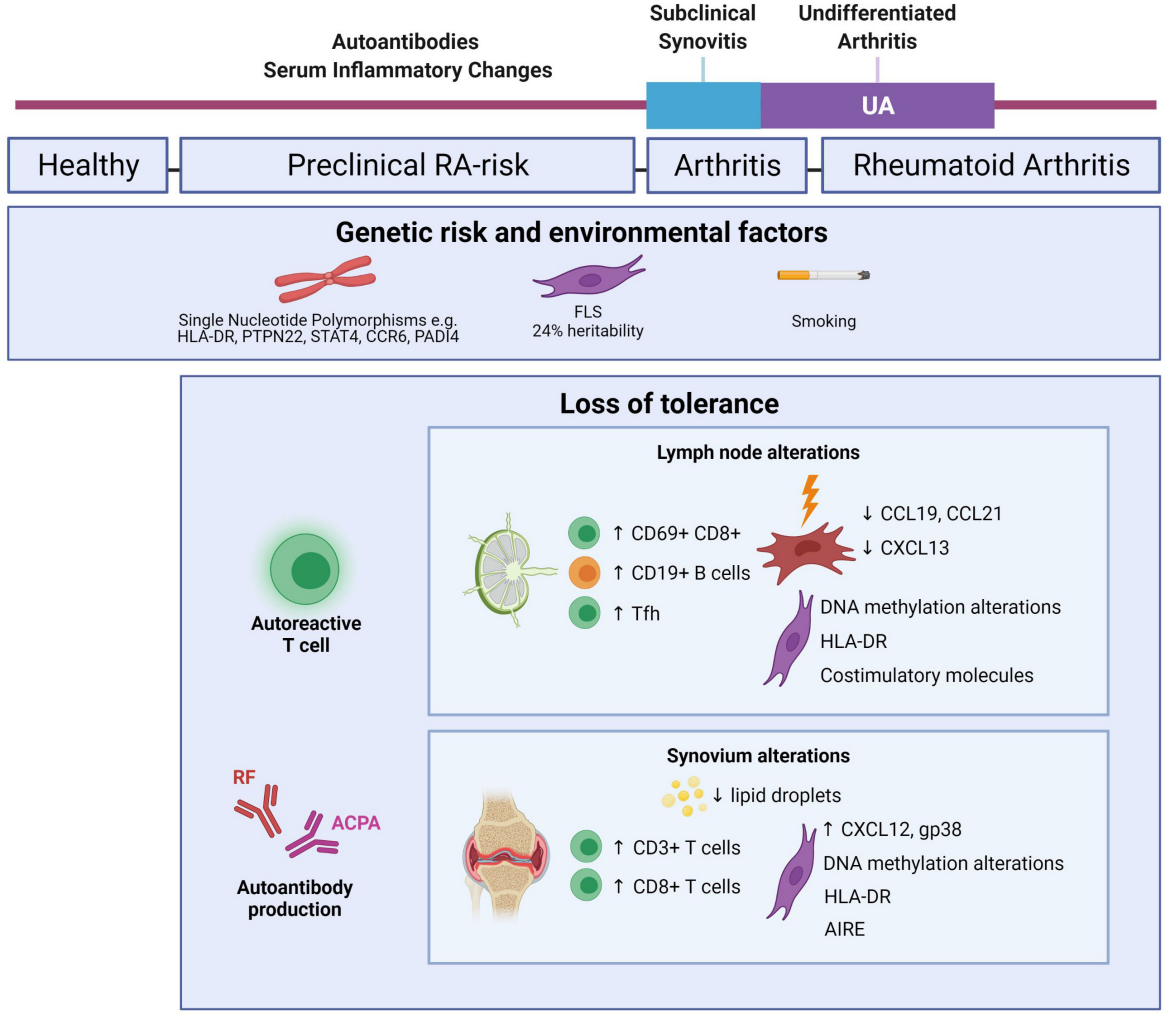
Mapping immune alterations during RA development. Schematic figure highlighting known changes within the immune system of RA-risk individuals and early RA. Genetic and environmental factors increase the likelihood of RA development in the healthy human population (5, 8). Depending on the risk profile, about 30% of RA-risk individuals go on to clinically develop RA (6). LN alterations are based on inguinal LN needle biopsy research comparing RA-risk individuals with healthy volunteers and early RA patients (9–13). Synovium alterations are based on synovial tissue biopsy research in RA-risk individuals and early RA patients (14–17, 57–59, 64, 65). Created with Biorender.com.

This review will outline our current knowledge of the immune cell interactions that occur in the LN of RA patients and how these may be linked to observations in the synovium (Figure 1). It will also postulate other immunological avenues that warrant future exploration to identify novel targets for treatment of early RA.

## LN Studies Reveal Immune Cell Activation During the Earliest Phases of RA

LNs are highly organized lymphoid structures situated throughout the human body that allow immune cell entry from surrounding tissues and blood to orchestrate a fast and effective immune response. They are the primary site of peripheral tolerance which aims to eliminate autoreactive T cells that escape central tolerance by exposing T cells residing in the LN to self-antigens. Studying human LNs during health and autoimmunity is challenging, because whole LNs are only obtained through surgery or autopsy and needle biopsies are too small to enable direct functional cellular analysis. The first study looking into LNs from RA patients suspected of lymphoma, only compared the cellular organization between LN and synovium in paired samples (17).

Our lab set up the infrastructure to study and compare LN needle biopsies from RA patients, RA-risk individuals and healthy volunteers (18). Initial cellular phenotyping by flow cytometry revealed that RA and RA-risk individuals have increased frequencies of CXCR3^+^CCR6^−^CCR4^−^ Th1 cells (19), ILC1 (c-Kit-NKp44^−^ ILCs) (20), memory CD8^+^ T cells (21), CD69^+^CD8^+^ T cells and more CD19^+^ B cells compared to healthy controls (9). Recent research suggests that CD69 expression may be indicative of a tissue resident memory T cell subset rather than an activated T cell subset (22). Evidence in mice suggests there is a circulating T effector population that is destined for a tissue resident phenotype (23, 24); however, the evidence for this in humans is undetermined. Of note, increased frequencies of CD69^+^CD8^+^ T cells were also observed in the blood of RA-risk individuals and RA patients (21). Whether the CD69^+^CD8^+^ T cells in LNs are tissue resident memory precursors that can later migrate and cause tissue inflammation within synovium warrants investigation.

T follicular helper cells (Tfh) are of particular interest as they can steer B cell activation and differentiation thereby potentially influencing autoantibody production. An increased frequency of both CD4^+^ and CD8^+^ Tfh could be detected in LN biopsies of RA-risk individuals and RA patients compared to healthy volunteers (10). Moreover, the augmented CD19^+^ B cell frequency in RA-risk individuals and RA patients correlated with Tfh frequency (9, 10). This may reflect germinal center activity resulting in autoantibody production within the LN due to break in tolerance, however, this needs further research.

Both dendritic cells (DCs) (25, 26) as well as lymph node stromal cells (LNSC) (27, 28) have the capacity to induce peripheral tolerance through presentation of peripheral tissue antigens (PTAs) to autoreactive T cells after which these cells undergo clonal deletion, differentiation into a regulatory T cell or become anergic. When studying DC subsets (CD1c^+^ myeloid DCs and CD304^+^ plasmacytoid DCs) in LN tissue it appeared that frequencies are comparable between RA-risk individuals and healthy controls, but increased in RA patients (29). This may suggest that these DCs are involved in sustaining inflammation during established RA, or that other DC subsets or other antigen presenting cells (APCs) are responsible for the initial break in tolerance. LNSC provide the structural integrity and framework for the important compartmentalization of lymphocytes within the LN (30). In recent years, mouse LNSC have been implicated more prominently in immune cell tolerance as they are able to delete self-reactive CD8^+^ T cells through PTA expression on major histocompatibility complex (MHC) I and on MHC II in the context of CD4^+^ T cells (27, 28, 31, 32). However, the presence of antibodies against self-antigens in early RA suggests this process is not always effective and provides impetus for examining LNSC in early RA. To investigate their functional capacities during RA development a human LNSC culture model has been developed (13). Transcriptome and methylome analysis of expanded LNSC highlighted key alterations in DNA methylation sites associated with antigen processing and presentation in RA-risk and RA patients when compared with healthy controls (11). Flow cytometry and qPCR data showed that all LNSC can express HLA-DR, co-stimulatory and co-inhibitory molecules (12), indicating that human LNSC are capable of modulating adaptive immunity. The expression of HLA-DR shows that human LNSC have the capacity to directly present PTAs while it was also shown that LNSC can express citrullinated antigens targeted by ACPAs (12). Whether such citrullinated antigens are presented as PTAs in LNSC and whether this process is altered in LNSC during systemic autoimmunity warrants further investigation.

LNSC produce key chemokines such as CCL19, CCL21 and CXCL13 to attract, retain and position lymphocytes within the LN and guide the interaction between T cells, B cells and APCs (33–35). Upon *in vitro* stimulation with TNFα and lymphotoxin α1β2, RA LNSC were significantly less capable to produce CCL19, CCL21 and CXCL13 (13). Furthermore, triggering TLR3 also showed lower induction of CCL19 in RA and RA-risk LNSC (36). Overall, these differences in RA-risk and RA LNSC may lead to imprecise localization of T cells upon LN entry and induce abnormal LNSC-T cell interactions leading to aberrant immune responses.

A remarkable feature observed in both RA and RA-risk LN T cells and LNSC is the diminished capacity to produce cytokines or chemokines upon *in vitro* stimulation which may indicate an exhausted phenotype (13, 19, 21, 36). T cell exhaustion is the result of repeated antigen stimulation which leads to increased inhibitory receptor expression such as PD1 and low effector function (37). Although extensively explored in chronic viral infection and cancer, the role of exhaustion in autoimmunity is as yet unclear. Future work to determine whether this reduction in cytokine production in LN T cells is a result of exhaustion is ongoing. Exhausted T cells are the target of immunotherapy such as anti-PD1 treatment. Recent studies of tumor draining LNs suggest they may harbor a reservoir of exhausted T cells that, if targeted, may improve therapeutic efficacy (38, 39). In the context of cancer, targeting an exhausted T cell population within the LN can allow for beneficial unleashing of the immune system. It is interesting to postulate whether exhaustion is induced in autoimmunity and how this may contribute to disease flare or remission. The increase in CD69^+^CD8^+^ T cells within RA and RA-risk LNs could also point toward a pre-exhausted memory phenotype. A recent human study identified a CD69^+^CXCR5^+^CXCR4^+^TCF1^+^ population in healthy LNs that had reduced expression of effector molecules and showed similarities to the mouse CD8^+^ T cell subset found to respond to checkpoint blockade therapy in a number of chronic infection mouse models (40). The CD69^+^CD8^+^ T cells increased in RA LNs may be an expansion of this newly identified human population which is possibly exhausted.

Overall, LN biopsy studies in RA-risk individuals provide a great model to explore the earliest break in tolerance which occurs in seropositive RA. Although, no LN data is currently available on the difference between RA-risk individuals that developed RA and those who did not, a number of changes observed in RA patients were also present in a proportion of RA-risk individuals. This may suggest a continuum that contributes to disease progression; however, follow up studies are needed to confirm this. Exploring if and how these early differences are reflected in the chronic inflammatory environment of the synovium provides great insight into early RA pathogenesis and may provide novel targets for preventive intervention.

## Insights From Synovium During Early RA Development

Several large cohort studies of RA synovium from treatment naïve patients have unearthed cellular heterogeneity. This enables pathology-based stratification which can be associated with clinical treatment response. The three main pathotypes described are diffuse myeloid, lympho-myeloid and pauci-immune representing myeloid dominance, lymphoid dominance and stromal dominance respectively (41–44). Analysis of synovial tissue biopsies not only allows for disease stratification and exploring how chronic inflammation persists in RA but also how it is initiated following loss of tolerance, presumably instigated in lymphoid organs. Synovial biopsies of autoantibody positive RA-risk individuals in almost all cases lack B cells and plasma cells (14) and do not yet display overt immune cell infiltration when compared to synovium from healthy controls (15), suggesting that synovial inflammation is likely to occur closer to clinical arthritis. The presence of CD3^+^ T cells in RA-risk synovium has been associated with subsequent arthritis development (14). Especially the combined presence of synovial CD8^+^ T cells with ACPA positivity increased the risk of RA development. This data suggest an early role for T cells in arthritis development. How these synovial T cells relate to the increased frequency of CD69^+^CD8^+^ T cells observed in the LNs of RA-risk individuals is worthy of exploration.

The origin of T cells in the synovium and how and where they have been activated is still largely unknown. Part of them may reflect activated resident T cells but probably the majority have been activated in peripheral lymphoid organs after which they then migrate to the synovium, as changes in the T cell compartment can be found in peripheral blood of early RA and RA-risk individuals (7, 45, 46). As previously highlighted, our work on LN-derived T cells suggest that there may be increased Tfh cells in RA patients (10) as well as a possibly exhausted population in RA-risk individuals (19, 21) which are both characterized by increased PD1 expression. Whether these changes are mirrored within the synovium is unclear. A number of studies have reported high PD1 expression on T cells within RA synovial tissue (47–49). Mass cytometry analysis of synovial tissue uncovered an expanded PD1^high^CXCR5^−^CD4^+^ T cell population that were akin to Tfh cells as they exhibited increased IL21, CXCL13, IFNγ and IL10 production (49). PD1^+^CXCR5^−^ memory CD4^+^ T cells were enriched within synovial tissue and fluid compared to their CXCR5^+^ memory CD4^+^ T cell counterpart; however, the phenotype of this population was not explored further (49). It was concluded that exhaustion was not present due to the population's ability to produce IL21 and CXCL13 (49). A larger, more in depth study is required to further characterize this PD1^high^CXCR5^−^ population and to determine at what phase of RA it emerges. Similar studies in juvenile idiopathic arthritis suggest that synovial T cells are effector populations and not exhausted (50); however, this has not been confirmed in the case of RA.

Not much is known about the role of CD8^+^ Tfh cells in RA, although the frequency of this population was also increased in RA LN (10). PD1^high^CD8^+^ T cells producing IL21 and also exhibiting increased CD28, ICOS, CD69 and HLA-DR expression have been observed in the synovial fluid and peripheral blood of RA patients (51) which like in the findings of Rao et al. (49) did also not express CXCR5. Whether this subset originates from the same CD69^+^CD8^+^ T cells found upregulated in the LN of RA-risk and early RA patients is of great interest but difficult to elucidate.

The possible interactions of these Tfh cells present within the synovium with B cells is interesting in relation to autoantibody production; however, B cells and plasma cells were not detectable by immunohistochemistry in synovial biopsies of RA-risk individuals (14, 15). This is despite the presence of autoantibodies and increased B cell frequencies in the lymph nodes of these individuals (9, 10). Furthermore, no significant changes have been observed in B cell frequency in the synovium of seropositive early RA compared to their seronegative counterpart (52) which further negates a role for B cells in autoantibody production within the synovium. Lymphoid neogenesis, relating to the aggregation of T and B cell lymphocytes, is observed in the synovium of a proportion of RA patients; however, its presence is associated with the degree of synovitis and not linked to clinical outcome or autoantibody status (53). This suggests these lymphoid aggregates are a result of chronic inflammation rather than a consequence of initial loss in peripheral tolerance.

It is clear from these studies that a population of T cells within the synovium that bear resemblance to Tfh cells and can provide B cell help are present in RA synovium. However, how this population arises and where these cells received their activation and differentiation cues is still contentious. Determining at what stage of RA pathogenesis this cell population arises will provide much insight into their functional role. Synovial cues directing the attraction, retention and activation of T cells may originate from altered resident synovial fibroblasts (FLS).

Fibroblasts in the synovium of patients with established RA have been studied for decades showing that these cells invade the cartilage and are responsible for tissue degradation and bone erosion [reviewed in (54)]. During destructive joint inflammation, FLS have an activated phenotype and secrete several immunomodulatory factors (55, 56). Epigenome and methylome analysis performed by several labs showed that FLS from RA patients undergo DNA methylation changes that can be linked to disease development (8, 57–59). A recent, elegantly designed functional genomics atlas study demonstrated that FLS account for up to 24% of RA heritability providing evidence of a causal role for FLS in RA development (8). This is in line with a prospective study which compared synovial biopsies of RA-risk individuals who later developed RA after follow up, with those who did not. Using immunohistochemistry analyses no overt immune cell infiltration was found in the synovium of RA-risk individuals who later developed disease (14, 15) while gene expression profiling points toward an activated stromal cell gene signature with increased podoplanin and CXCL12 levels and decreased lipid droplets (16). These studies provide strong evidence for a causal role for FLS in driving disease pathogenesis at an early stage.

Fibroblasts are known to exhibit immunomodulatory capacities when interacting with nearby or incoming immune cells and several studies have investigated their potential as antigen-presenting cells (60, 61). Similar to stromal cells in the LN, FLS create a pro-survival and anti-apoptotic microenvironment in the synovium by secreting cytokines and chemokines that support the survival of immune cells supporting inflammation (62, 63). AIRE was recently identified as a cytokine-induced RA-risk gene in RA FLS (64). RNA sequencing revealed that AIRE did not induce PTAs in FLS, but stimulates pro-inflammatory cytokine and chemokine secretion associated with RA development. AIRE expression was hardly detected on mRNA level in unstimulated RA FLS, but largely increased and detected at protein level after TNFα and IL1β stimulation (64). Single-cell RNA sequencing on RA FLS showed a putative subpopulation of CD90^+^HLA-DR^high^ FLS expanded in the sublining of RA synovium. Additionally, inflammatory mediators such as IL6 could be linked to this FLS subset (65). The HLA-DR^high^ FLS subpopulation was shown to express genes related to MHC-II presentation and IFNγ signaling (65), suggesting that FLS have the capacity to present antigens and interact with immune cells directly. It will be of interest to investigate whether this HLA-DR^high^ FLS subset is expanded already in RA-risk individuals and to study its potential capacity to regulate peripheral tolerance.

## Future Research Avenues

**Figure 2:**
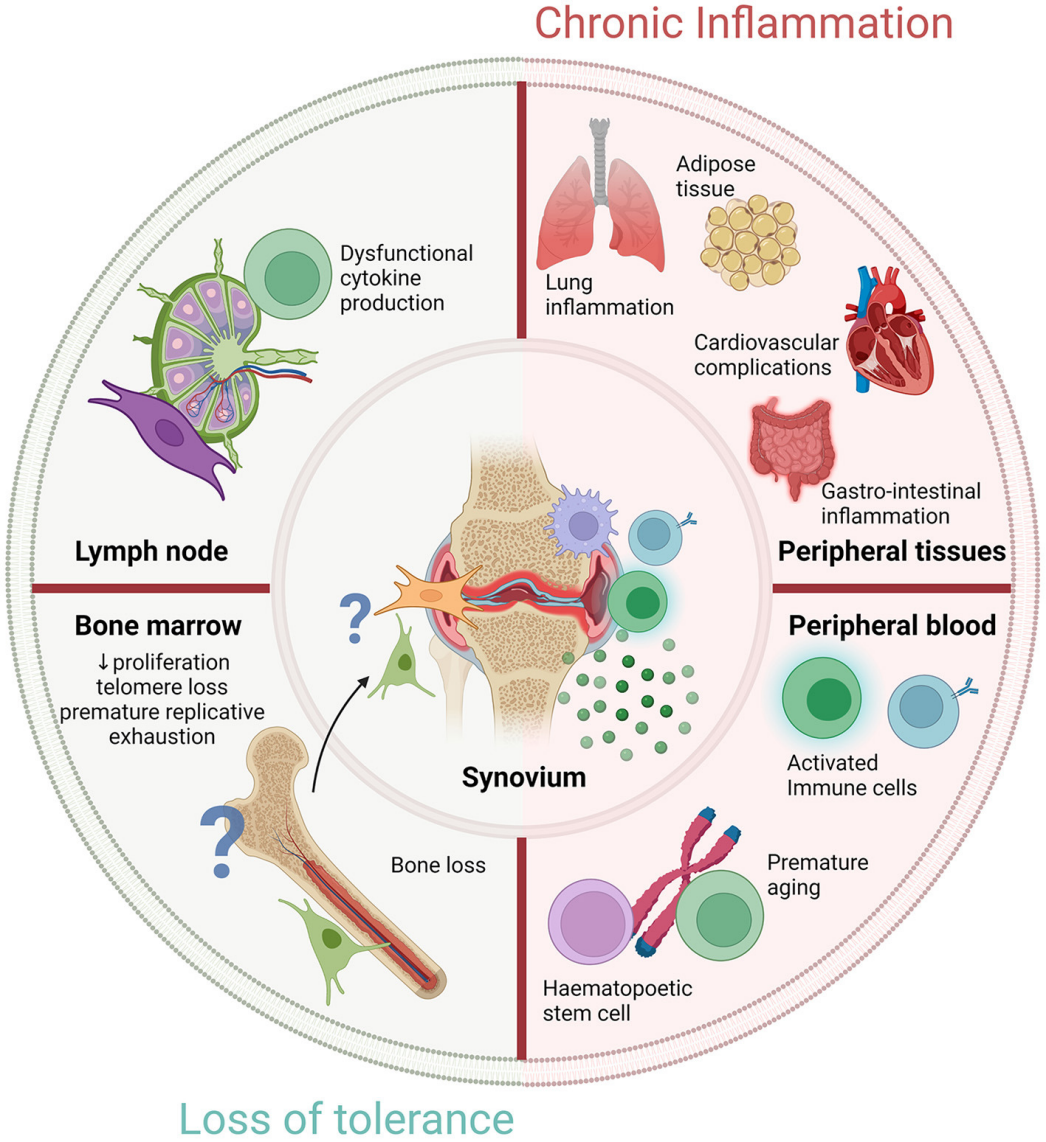
Novel targets to restore cellular fitness before RA development. Diagram representing novel targets of either chronic inflammation or loss of tolerance to prevent RA development in RA-risk individuals. Outer circle shows four critical areas that show significant changes in early RA. Lymph node and bone marrow alterations are observed, potentially due to a loss of tolerance in RA which warrant further investigation. Timely normalization of cellular fitness within lymphoid organs or other peripheral tissues could possibly prevent RA development in RA-risk individuals. Peripheral blood research may identify predictive biomarkers for early diagnosis or disease prognosis. Chronic inflammation is observed in distinct locations outside the synovium but whether these are the consequence of a loss of tolerance or caused by inflammation is unknown. The locations of the outer circle culminate in the chronic inflammatory environment observed within the synovium but how exactly they contribute warrants further examination. Created with Biorender.com.

Detailed analysis of tissues involved in initial triggering of RA are fundamental for finding predictive biomarkers for disease progression and for the discovery of novel drug targets to aid future preventive treatment strategies (Figure 2). As said, we propose that the first signs of RA can be identified in lymphoid organs as they are the epicenter of immune activation in which APCs, including stromal cells, can trigger T-cell mediated B-cell activation, thereby initiating an inflammatory response and autoantibody production. Studying tissue samples derived from lymphoid organs of individuals at risk of developing RA will provide crucial insights into the earliest stages of disease pathogenesis. Presently, within the field of rheumatology, only a few studies have been performed on human LN biopsies while this biopsy procedure is frequently performed in routine clinical care in the field of oncology. In addition, our observational study investigating the perspectives of study participants, indicated that inguinal LN biopsy sampling is well-tolerated, safe and provides sufficient material for further molecular and cellular analyses (Fiechter et al.)^1^. Accordingly, available data provides a strong case for the application of this research tool in order to identify novel biomarkers and drug targets in individuals at risk of developing RA.

A so far unexplored lymphoid organ in RA-risk individuals is the bone marrow (BM), while already decades ago it has been postulated that RA may be a BM disorder (66). A more recent micro-CT study has shown bone loss in ACPA positive healthy individuals without clinical signs of arthritis (67). Of interest, animal labeling studies have shown migration of fibroblastic cells from the BM to the synovium at the onset of arthritis (68). Besides the production of hematopoietic stem cells, the BM is highly important for the development of mesenchymal stromal cells (MSC). There is compelling evidence that BM-MSC are instrumental for effective hematopoiesis and have important immunomodulatory and regenerative capacities (69–71). Accordingly, clinical trials have been initiated to examine the therapeutic properties of MSC in many different diseases, including RA (72). However, the inconsistent findings reported, reflect that the understanding of MSC biology, especially in the context of autoimmunity, is limited. A few studies have investigated BM-MSC in patients with RA (73–75). These studies show that RA BM-MSC are normal in frequency and have a normal differentiation capacity (73). However, RA BM-MSC exhibit reduced proliferation, telomere loss, and premature replicative exhaustion (73). Moreover, BM-MSC from RA patients are less supportive for hematopoietic stem and progenitor cell (HSPC) survival (74). Currently, it is unknown whether these BM-MSC abnormalities are caused by inflammation or take place already before disease onset in RA-risk individuals.

Increased immune cell aging, determined by measuring the shortening of telomeres, has been observed in several chronic inflammatory diseases, including RA (76–78). Immune cells from 30 year-old RA patients show a biological cellular age of a 50 year-old healthy individual (79). Of note, in RA this accelerated aging is already observed in HSPC (80); however, it is unknown whether premature aging is an intrinsic defect present already before onset of disease or a secondary effect due to inflammatory signals or treatment. It is unclear whether this accelerated aging originates in lymphoid organs and is also observed in tissue resident cells like MSC which will have a major influence on MSC-mediated cell survival and immunomodulation.

Many of the questions still outstanding regarding RA pathogenesis are those of disease origin and cellular contribution. We postulate that RA-risk individuals can be studied as a model to investigate the earliest phases of systemic autoimmunity wherein self-tolerance is lost potentially due to accelerated cellular aging affecting adaptive immune responses. It is important to unravel whether accelerated or premature aging can already be detected in preclinical phases of RA by studying cellular characteristics of senescence in lymphocytes as well as stromal cells isolated from synovial, LN and BM tissue biopsies obtained from RA-risk individuals. If present, such research will lead to the discovery of novel targets to restore cellular fitness before RA onset. Research focusing on detailed analyses of human tissue samples obtained during the earliest preclinical phases of RA are highly challenging but will provide great advancement toward the ultimate goal of disease prevention.

## Data Availability Statement

The original contributions presented in the study are included in the article/supplementary material, further inquiries can be directed to the corresponding author/s.

## Author Contributions

All authors listed have made a substantial, direct, and intellectual contribution to the work and approved it for publication.

## Funding

LB received an AMC Fellowship and funding from a ZonMw VIDI project (91718371) and from the European Union's Horizon 2020 Research and Innovation Program under the Marie Skłodowska-Curie grant agreement No. 847551 (ARCAID).

## Conflict of Interest

The authors declare that the research was conducted in the absence of any commercial or financial relationships that could be construed as a potential conflict of interest.

## Publisher's Note

All claims expressed in this article are solely those of the authors and do not necessarily represent those of their affiliated organizations, or those of the publisher, the editors and the reviewers. Any product that may be evaluated in this article, or claim that may be made by its manufacturer, is not guaranteed or endorsed by the publisher.

## Footnotes

^1^Fiechter RH, Bolt JW, van de Sande MGH, Aalbers CJ, Landewé RBM, Maas M, et al. Ultrasound-guided lymph node biopsy sampling to study the immunopathogenesis of rheumatoid arthritis: a well-tolerated valuable research tool.

## Abbreviations

RA: rheumatoid arthritis
RA-risk: individuals at risk of developing rheumatoid arthritis
LN: lymph node
LNSC: lymph node stromal cells
FLS: synovial fibroblasts
BM: bone marrow
BM-MSC: bone marrow stromal cells
PTA: peripheral tissue antigens
DC: dendritic cell
APC: antigen presenting cell
Tfh: T follicular helper
IL21: Interleukin 21
RF: rheumatoid factor
ACPA: anti-citrullinated peptide antibodies.
